# The Efficacy and Safety of Epidermal Growth Factor Receptor Tyrosine Kinase Inhibitor Combined With Thymosin in Advanced Non-Small Cell Lung Cancer Patients Harboring Active Epidermal Growth Factor Receptor Mutations

**DOI:** 10.3389/fonc.2021.659065

**Published:** 2021-05-28

**Authors:** Yongdong Feng, Guangkuo Zhu, Song Lang, Ping Hao, Guanghui Li, Fanglin Chen, Wenlei Zhuo, Yuzhong Duan, Anmei Zhang, Zhengtang Chen, Jianguo Sun

**Affiliations:** Cancer Institute, Xinqiao Hospital, Army Medical University, Chongqing, China

**Keywords:** NSCLC, EGFR-TKI, thymosin, active EGFR mutations, efficacy, safety

## Abstract

**Objective:**

To explore the efficacy and safety of EGFR-TKI combined with thymosin therapy in advanced non-small cell lung cancer (NSCLC) patients harboring active EGFR mutations.

**Methods:**

Patients confirmed as advanced NSCLC with active EGFR mutations were recruited from August 2008 to July 2018 retrospectively. Patients treated with EGFR-TKI were classified as the EGFR-TKI group. And those received EGFR-TKI and thymosin therapy were designated as the EGFR-TKI plus thymosin group. The primary endpoint was progression-free survival (PFS). The secondary endpoints included overall survival (OS), tumor response and adverse effects.

**Results:**

The median PFS was significantly longer in EGFR-TKI plus thymosin group than that in EGFR-TKI group (14.4 months *vs*. 9.2 months; HR=0.433, 95% CI 0.322 - 0.582, *P*<0.0001). The median OS was also prolonged in EGFR-TKI plus thymosin group than that in EGFR-TKI group (29.5 months *vs*. 19.8 months; HR=0.430, 95% CI 0.319 - 0.580, *P*<0.0001). The objective response rate in EGFR-TKI plus thymosin group and EGFR-TKI group were 60.0% versus 60.8% (*P*=0.918). The disease control rate was 96.9% in EGFR-TKI plus thymosin group and 97.7% in EGFR-TKI group (*P*=1.000). There were no significant differences in adverse effects between the two groups. The number of CD3^+^T cells in peripheral blood decreased significantly after treatment including both CD3^+^CD4^+^T and CD3^+^CD8^+^T subsets in EGFR-TKI group, but not in EGFR-TKI plus thymosin group.

**Conclusions:**

Combination of EGFR-TKI and thymosin can significantly prolong the PFS and OS compared with EGFR-TKI monotherapy without more adverse events, which offers a new strategy in clinic.

## Introduction

Epidermal growth factor receptor tyrosine kinase inhibitor (EGFR-TKI) is currently recommended as a standard first-line therapy for advanced non-small cell lung cancer (NSCLC) patients harboring active EGFR mutations, which was reported to prolong progression-free survival (PFS) compared with standard platinum-based chemotherapy significantly (1–3). However, most of the NSCLC patients with an initial dramatic response to EGFR-TKI treatment developed progression disease after 8.40-13.10 months (4, 5). In order to prolong the survival time of NSCLC patients with active EGFR mutations, novel drugs including osimertinib and crizotinib were developed by targeting resistance mechanisms (6, 7). Besides, combination therapies, such as EGFR-TKI combined with angiogenesis inhibitorwas shown to improve the PFS (8), but not OS (9). EGFR-TKI combined with chemotherapy prolonged PFS and OS but increased toxicity significantly (10). At present, there is still no satisfactory combination therapy.

As reported, sensitive EGFR-TKIs caused obvious tumor microenvironmental changes including the number of immune cells and inflammatory factors in serum (11). EGFR blockade by using erlotinib reduced CD4^+^T cell proliferation in response to soluble anti-CD3 stimulation (12). Erlotinib demonstrated an immunosuppressive activity on T-cell-mediated immune response both *in vitro* and *in vivo* (13). Therefore, the combination of immunomodulators may be a potential method to enhance the efficacy of EGFR-TKI. In clinic, thymosin such as thymosin alpha 1 and thymopentin, have been widely used as immunomodulators in kinds of cancers (14). As reported, thymosin can significantly improve patient’s quality of life by enhancing T-cell function, stimulation of T cell maturation and differentiation in lung cancer (15). In addition to the effect on immunomodulatory, thymosin has been reported to exert synergistic antitumor activity without more adverse effects when combined with chemotherapy in lung cancer (16). Whether thymosin combined with EGFR-TKI can improve patients’ PFS and OS needs to be illustrated.

In this study, we conducted a retrospective study to explore the efficacy and safety of EGFR-TKI plus thymosin in NSCLC patients harboring active EGFR mutations, thereby enhancing the efficacy of EGFR-TKI.

## Materials and Methods

### Patient Population

We conducted a retrospective research during August 2008 to July 2018 in three Affiliated Hospitals of Army Medical University (Chongqing, China). Patients over the age of 18 years were histologically or cytologically confirmed as stage IV NSCLC were recruited. TNM classification (tumor, node, and metastasis) of lung cancer was made according to the American Journal of Critical Care (AJCC) 7th edition of Lung Cancer. Those patients with active EGFR mutations, including exon 19 deletion or exon 21 L858R mutation, were detected by amplification refractory mutation system or next-generation sequence. Patients received first or second-line EGFR-TKI therapy, whose Eastern Cooperative Oncology Group (ECOG) performance status (PS) score were 0-2, had one or more measurable target lesions and follow-up time >3 months were included. Exclusion criteria included incomplete medical records, EGFR-TKI treatment less than 8 weeks and thymosin therapy less than 4 weeks, loss to follow-up. This trial had been approved by the Ethics Committee, Xinqiao Hospital, Army Medical University (2018–302–01). All procedures were conducted in accordance with the Declaration of Helsinki.

### Treatment

Patients treated with EGFR-TKI (erlotinib 150mg daily, gefitinib 250mg daily or icotinib 125mg three times a day) alone were classified as the EGFR-TKI group. Patients received EGFR-TKI (erlotinib 150mg daily, gefitinib 250mg daily or icotinib 125mg three times a day) and thymosin (thymosin α1 1.6mg twice a week or thymopentin 10mg daily) concurrently were designated as the EGFR-TKI plus thymosin group.

### Data Collection

The data collected included the demographics, clinical characteristics, treatment, tumor response, adverse events (AEs) and T lymphocyte subsets in peripheral blood. Tumor response was assessed by two independent senior physicians in oncology according to RECIST 1.1. The initial response was assessed after 4 weeks of treatment, and tumor evaluation was repeated every 2 months. If the assessments were inconsistent, the controversial results were reassessed by the third oncologist. AEs were assessed according to the National Cancer Institute Common Toxicity Criteria version 3.0. The data of peripheral blood T lymphocyte subsets before EGFR-TKI treatment were gathered within one month before EGFR-TKI treatment. Peripheral blood T lymphocyte subsets after EGFR-TKI treatment were collected from receiving EGFR-TKI treatment to progressive disease, and data after EGFR-TKI plus thymosin treatment were acquired from patients receiving EGFR-TKI plus thymosin treatment to progressive disease, and then taking the average of all these data. 2mL of venous blood was collected in EDTA anticoagulant tube from each patient. Peripheral blood T lymphocytes subsets were detected by flow cytometry by trained technicians in Clinical Laboratory of Xinqiao Hospital in Army Medical University. The samples were stained with antibody to human CD45, CD3, CD4, CD8 or isotype control conjugated with PerCP, FITC, APC and PE for 30 min, respectively. The indicated antibodies were obtained from Agilent Technologies (China Inc). Subsequently, stained samples were measured on a flow cytometer, NovoCyte D2040R (Agilent Technologies). The data were analyzed by NovoExpress software (Agilent Technologies).

### Assessment Criteria

The primary endpoint was PFS, which was defined as the time from the initiation of EGFR-TKI treatment to the first documentation of progressive disease or death for any reason. The secondary endpoints included OS, objective response rate (ORR), disease control rate (DCR) and AEs. OS was defined as the time from the first dose of EGFR-TKI to cancer-related death or the last follow-up time.

### Statistical Analysis

When comparing the baseline, brain metastasis, bone metastasis and first- or second-line EGFR-TKI treatment had significantly differences in EGFR-TKI group and EGFR-TKI plus thymosin group. Propensity score matching was applied at the ratio of 2:1 in EGFR-TKI group and EGFR-TKI plus thymosin group with these three items as covariates to avoid bias. The Chi-square test or fisher exact test was used to analyze the intergroup difference in clinical features, ORR, DCR and AEs. And the Wilcoxon rank sum test was used to analyze age difference. The Kaplan-Meier estimator was used to estimate the survival curves. The log-rank test was performed for intergroup comparison. Cox proportional hazards regression was used to calculate hazard ratios (HRs) and 95% confidence intervals (CIs) for the association of combination of EGFR-TKI and thymosin with the risk of disease progression and death. The changes of peripheral blood T lymphocyte subsets between EGFR-TKI and EGFR-TKI plus thymosin group before and after treatment were assessed by paired-samples T test. All statistical analyses were performed using R software (Version 3.6.1). The statistical significance level was set at *P* < 0.05 under a two-tailed test.

## Results

### Patient Characteristics

From August 2008 to July 2018, a total of 908 patients were confirmed as NSCLC with active EGFR mutations. Of these patients, 495 subjects met the inclusion criteria. There were 22 patients excluded for incomplete medical records. 36 cases of the patients received EGFR-TKI therapy for less than 8 weeks. 89 patients were treated with thymosin for less than 4 weeks. And there were 16 patients lost to follow-up. Finally, 267 patients received EGFR-TKI monotherapy and 65 patients received EGFR-TKI plus thymosin treatment (**Supplementary Figure 1**).

When conducted the comparison of demographic and clinical characteristics between EGFR-TKI group and EGFR-TKI plus thymosin group, we found that brain metastasis, bone metastasis and first- or second-line EGFR-TKI treatment in two groups had significantly differences, then propensity score matching analysis was applied at a ratio of 2:1. Finally, 130 patients took EGFR-TKI monotherapy and 65 patients received EGFR-TKI plus thymosin treatment were included (**Supplementary Figure 1**). Of the 195 patients, the median age was 57.5 years old (range, 21 to 81 years old) and 120 cases were female. All of them were diagnosis as adenocarcinoma histologically. There were 144 patients received EGFR-TKI as the first line therapy and 51 patients received the second line therapy. 93 patients received gefitinib therapy, 76 patients took received erlotinib therapy and 26 patients were treated with icotinib. The baseline of EGFR-TKI group and EGFR-TKI plus thymosin group were balanced after propensity score matching (**Table 1**).

**Table 1:** Patients’ demographic and clinical characteristics in the EGFR-TKI and EGFR-TKI plus thymosin group before and after propensity score matching.

Characteristics	Before match			After match		
	EGFR-TKI n=267	EGFR-TKI plus thymosin n=65	*P*	EGFR-TKI n=130	EGFR-TKI plus thymosin n=65	*P*
Male	104	23	0.596	52	23	0.532
Median age (IQR)	57 (49-64)	58 (49-66)	0.357	57 (48-65)	58 (49-66)	0.489
ECOG PS						
0-1	260	61	0.236	127	61	0.225
2	7	4		3	4	
Smoking	65	11	0.202	34	11	0.149
EGFR mutations						
Exon 19 deletion	156	38	0.865	78	38	0.982
Exon 21 L858R mutation	112	26		53	26	
CNS metastasis	82	11	0.026	22	11	1.000
Liver metastasis	24	9	0.241	13	9	0.424
Multiple lung metastasis	136	28	0.256	69	28	0.188
Pleura metastasis	98	19	0.258	46	19	0.390
Bone metastasis	123	46	0.0004	92	46	1.000
Adrenal metastasis	17	8	0.104	8	8	0.140
EGFR-TKI						
Gefitinib	133	29	0.258	64	29	0.258
Erlotinib	96	30		46	30	
Icotinib	38	6		20	6	
Line of EGFR-TKI						
First-line	219	46	0.043	98	46	0.489
Second-line	48	19		32	19	
Radiotherapy for CNS metastasis	18	7	0.270	8	7	0.254
Radiotherapy for lung lesion	4	3	0.139	2	3	0.336
Radiotherapy for bone metastasis	10	6	0.100	6	6	0.219

EGFR, epidermal growth factor receptor; TKI, tyrosine kinase inhibitor; IQR, interquartile range; ECOG PS, eastern cooperative oncology group performance status; CNS, central nervous system.

### Progression-Free Survival and Overall Survival

**Figure 1:**
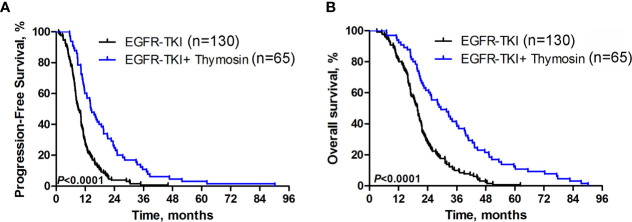
Progression-free Survival and Overall Survival. Kaplan-Meier estimated **(A)** progression-free survival (PFS) and **(B)** overall survival (OS) in EGFR-TKI plus thymosin group and EGFR-TKI group.

The median PFS was 14.4 months (95% CI, 11.7-17.1) in EGFR-TKI plus thymosin group, which was significantly improved than 9.2 months (95% CI, 7.9-10.3) in EGFR-TKI group (HR=0.433, 95% CI 0.322 - 0.582, *P*<0.0001, **Figure 1A**). The median OS were 29.5 months (95% CI, 21.5-37.5) in EGFR-TKI plus thymosin group and 19.8 months (95% CI, 18.2-21.4) in EGFR-TKI group, respectively (HR=0.430, 95% CI 0.319 - 0.580, *P*<0.0001, **Figure 1B**).

**Figure 2:**
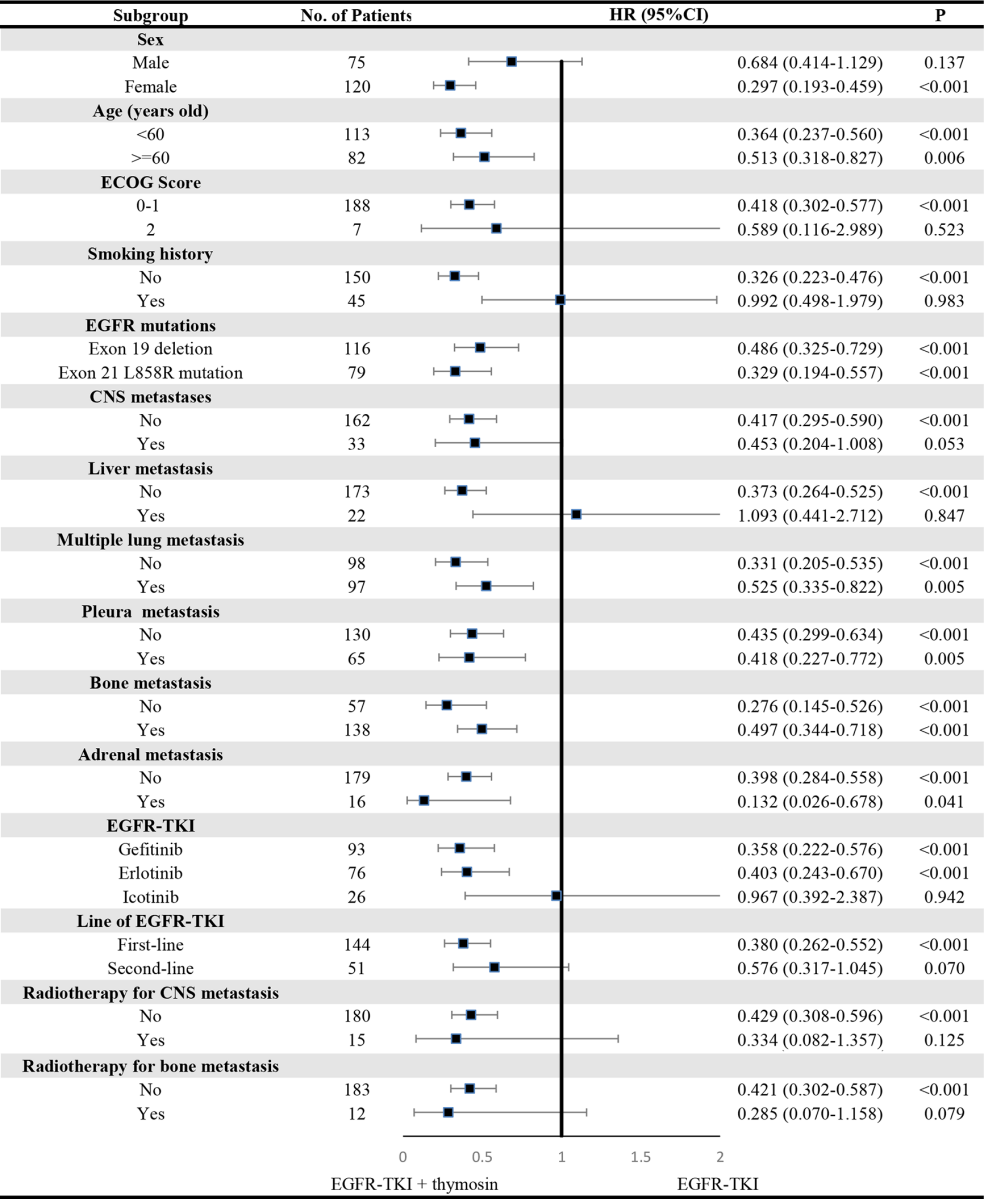
Subgroup Analyses of Progression-free Survival. P showed the significance of the HRs. It was used to test the significance of PFS between EGFR-TKI plus thymosin group and EGFR-TKI group in a certain variable. HR, hazard ratio; CI, confidence interval; ECOG, eastern cooperative oncology group; CNS, central nervous system.

**Figure 3:**
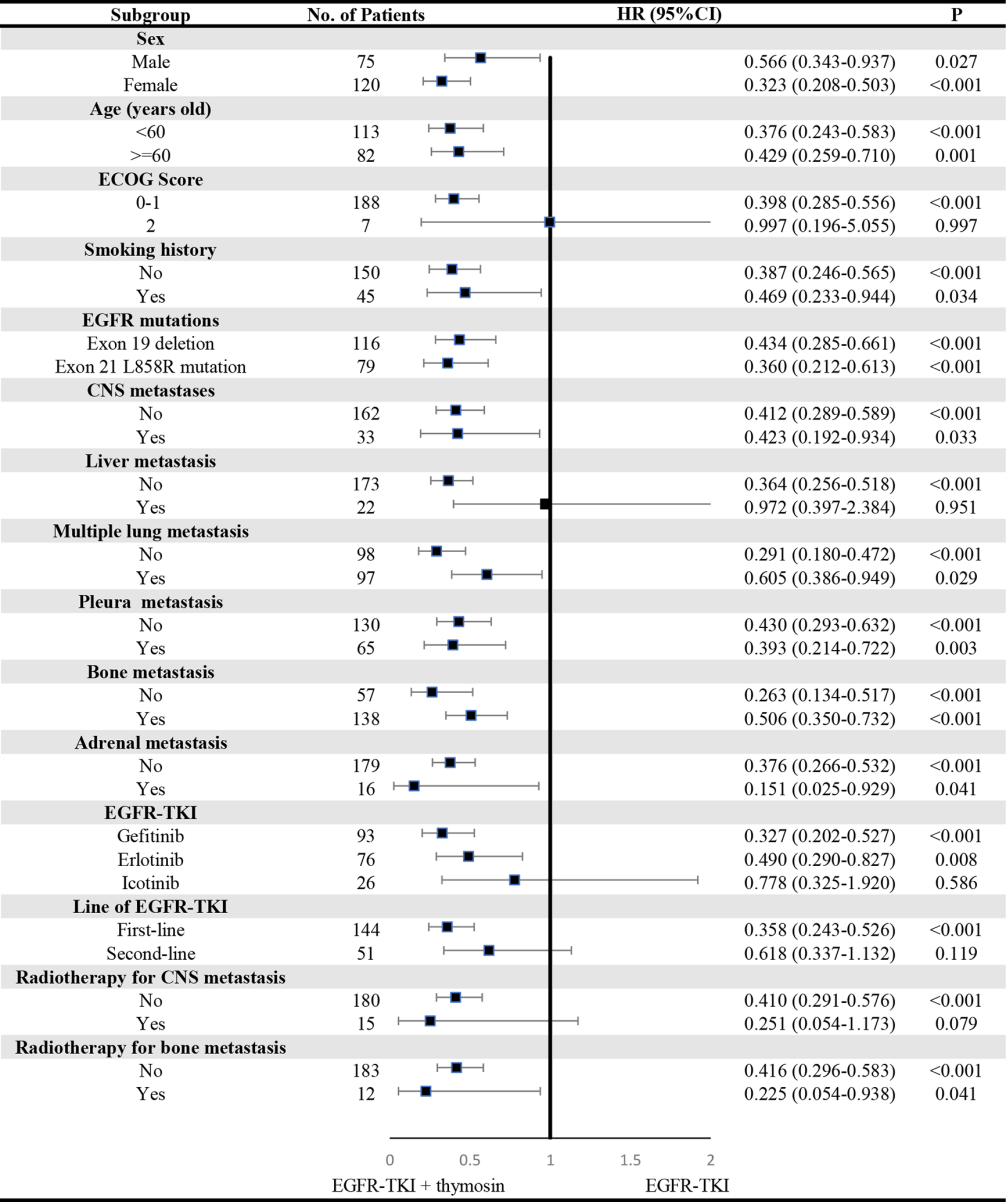
Subgroup Analyses of Overall Survival. P showed the significance of the HRs. It was used to test the significance of OS between EGFR-TKI plus thymosin group and EGFR-TKI group in a certain variable. HR, hazard ratio; CI, confidence interval. ECOG, eastern cooperative oncology group; CNS, central nervous system.

A consistent benefit of EGFR-TKI plus thymosin over EGFR-TKI with respect to PFS (**Figure 2**) and OS (**Figure 3**) were shown across most subgroups that were assessed, including the subgroups based on age, EGFR mutation type (exon 19 deletion vs. exon 21 L858R mutation), the presence or absence of CNS metastases, multiple lung metastasis, pleura metastasis, bone metastasis and adrenal metastasis, treated by gefitinib or erlotinib. The advantages of PFS in EGFR-TKI plus thymosin group were not observed in males, patients whose ECOG score was 2 points, who had smoking history, suffered from liver metastasis, treated with icotinib, received the EGFR-TKI as the second line therapy and received radiotherapy (**Figure 2**). And OS in EGFR-TKI plus thymosin group had no advantage in patients who suffered from liver metastasis, treated with icotinib, received the EGFR-TKI as the second line therapy. Patients who received radiotherapy for CNS metastasis had a tendency to benefit from the combination therapy (*P*=0.079) (**Figure 3**).

In EGFR-TKI plus thymosin group, there were 29 patients treated with EGFR-TKI plus thymosin α1 whose median PFS were 14.7 months and 36 patients treated with EGFR-TKI plus thymopentin with the median PFS of 14.1 months (*P*>0.05). And there was no difference in the median OS between the two groups.

### Response Rates

The objective response rate (ORR) was 60.0% in EGFR-TKI plus thymosin group and 60.8% in EGFR-TKI group (*P*=0.918). The disease control rate (DCR) was 96.9% in EGFR-TKI plus thymosin group and 97.7% in EGFR-TKI group (*P*=1.000). There were no differences in ORR and DCR between the two groups. More details about response rates were shown in **Supplementary Figure 2**.

### Adverse Events

The most common adverse events were rash (40.0% in the EGFR-TKI plus thymosin group and 38.5% in the EGFR-TKI group), diarrhea (23.1% and 25.4%, respectively), dry skin (20.0% and 20.8%, respectively) and anorexia (9.2% and 10.0%, respectively). Adverse events of grade 3 or higher occurred in 2 cases in the EGFR-TKI plus thymosin group and 5 cases in the EGFR-TKI group. No fatal adverse events were found in the two groups. There were no significant differences in adverse effects between the two groups (**Table 2**). Together, the combination of EGFR-TKI and thymosin would not increase the incidences of adverse events.

**Table 2:** Adverse effects in EGFR-TKI and EGFR-TKI plus thymosin group.

Adverse effect	EGFR-TKI n=130 (%)		EGFR-TKI plus Thymosin n=65 (%)		*P*
	ALL grades	Grades≥3	ALL grades	Grades≥3	
Rash	50 (38.5)	3 (2.3)	26 (40.0)	1 (1.5)	0.836
Diarrhea	33 (25.4)	2 (1.5)	15 (23.1)	1 (1.5)	0.724
Dry skin	27 (20.8)	0 (0.0)	13 (20.0)	0 (0.0)	0.900
Hand and foot syndrome	0 (0.0)	0 (0.0)	1 (1.5)	0 (0.0)	0.333
Tired	6 (4.6)	0 (0.0)	1 (1.5)	0 (0.0)	0.276
Anorexia	13 (10.0)	0 (0.0)	6 (9.2)	0 (0.0)	0.864
Nausea,vomiting	2 (1.5)	0 (0.0)	2 (3.1)	0 (0.0)	0.602
Increasing of transaminase	4 (3.1)	0 (0.0)	0 (0.0)	0 (0.0)	0.303
Mouth ulcer	6 (4.6)	0 (0.0)	0 (0.0)	0 (0.0)	0.181
Nail changes	0 (0.0)	0 (0.0)	1 (1.5)	0 (0.0)	0.333
Reduction of leukocyte	1 (0.8)	0 (0.0)	1 (1.5)	0 (0.0)	1.000
Deadlimb	0 (0.0)	0 (0.0)	1 (1.5)	0 (0.0)	0.333
Edema	0 (0.0)	0 (0.0)	1 (1.5)	0 (0.0)	0.333

EGFR, epidermal growth factor receptor; EGFR-TKI, epidermal growth factor receptor tyrosine kinase inhibitor.

### Peripheral Blood T Lymphocyte Subsets

**Figure 4:**
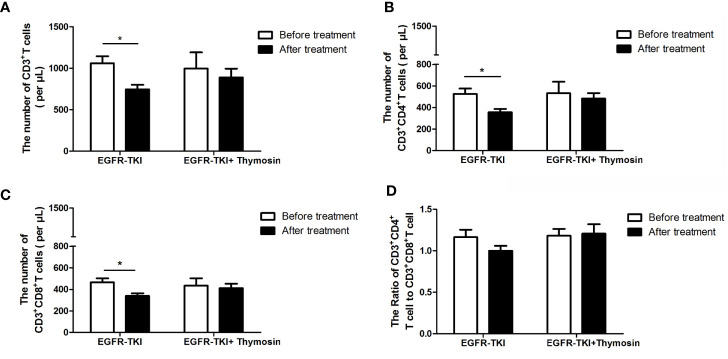
The numbers of T cell subsets in peripheral blood in EGFR-TKI group and EGFR-TKI plus thymosin group before and after treatment. **(A)** The numbers of CD3^+^ T cells. **(B)** The numbers of CD3^+^ CD4^+^T cells. **(C)** The numbers of CD3^+^ CD8^+^T cells. **(D)** The ratio of CD3^+^ CD4^+^T cells to CD3^+^ CD8^+^T cells. *P < 0.05.

There were 23 patients detected the peripheral blood T lymphocyte subsets before and after EGFR-TKI monotherapy, and 11 patients had detected the peripheral blood T lymphocyte subsets before and after EGFR-TKI plus thymosin therapy. In the EGFR-TKI group, the number of CD3^+^T cells decreased after treatment (*P*<0.05, **Figure 4A**) including both the CD3^+^CD4^+^T and CD3^+^CD8^+^T subsets (*P*<0.05, **Figures 4B, C**). However, the ratio of CD3^+^CD4^+^T to CD3^+^CD8^+^T did not change (**Figure 4D**). In the EGFR-TKI plus thymosin group, both the number of CD3^+^T cells and the ratio of CD3^+^CD4^+^T to CD3^+^CD8^+^T had no obvious changes before and after treatment (**Figure 4**). It suggested that thymosin combined with EGFR-TKI may reverse the inhibition of T cells.

## Discussion

In the current study, our results demonstrated that patients in EGFR-TKI plus thymosin group had significantly prolonged median PFS and OS compared with those in EGFR-TKI group. And the combination of EGFR-TKI and thymosin would not increase the incidences of adverse events. By observing the peripheral blood T lymphocyte subsets before and after treatment, we found thymosin combined with EGFR-TKI may reverse the inhibition of T cells. Therefore, combination of EGFR-TKI and thymosin may be a potential therapy to enhance the efficacy of EGFR-TKI without increasing adverse events.

To avoid the impact of inconsistent baseline, the propensity score matching analysis was applied at the ratio of 2:1 in EGFR-TKI group and EGFR-TKI plus thymosin group. The limitation of propensity score matching analysis is only to control the effect of measurable variables. New bias may appear if there is unobservable selection on variables. However, the results will be less reliable when the confounding factors exist. In this study, there were no differences in demographic and clinical characteristics between the two groups after propensity score matching.

The median PFS in EGFR-TKI plus thymosin group was 14.4 months, with a 57% lower risk of disease progression or death than that in the EGFR-TKI group. The median PFS in the EGFR-TKI group in our research is 9.2 months which is similar with that in previous clinical trials of gefitinib with the median PFS ranges from 8.0 (5) to 10.9 (17) months, erlotinib with the median PFS 13.1 months (4) and icotinib with the median PFS 11.2 months (18). As previously reported, the median OS of EGFR-TKIs (including gefitinib, erlotinib and icotinib) ranged from 21.6 (19) to 30.5 months (2, 18), which was longer than the OS in our study. Zeng et al. (16) had summarized four trials with 269 cases to demonstrate that administration of thymosin with chemotherapy significantly increased the 1-year OS rate. Our study had also showed that combination of EGFR-TKI and thymosin could prolong the PFS and OS and would be a potential therapy to enhance the efficacy of EGFR-TKI.

In the subgroup analysis, consistent benefit of EGFR-TKI plus thymosin over EGFR-TKI with respect to PFS and OS were shown. Patients benefit from EGFR-TKI plus thymosin treatment regardless of the type of EGFR mutations. Fukuoka et al. (19) had reported that PFS was significantly longer for gefitinib versus carboplatin/paclitaxel in both the exon 19 deletions and the exon 21 L858R mutation subgroups. Combined EGFR-TKI with thymosin was more advantageous as first-line treatment compared with who received it as second-line treatment, which was consistent with the result of EGFR-TKI monotherapy (2). OS in EGFR-TKI plus thymosin group had no advantage in patients who suffered from liver metastasis. No comparison stratified by liver metastasis was retrieved in studies about EGFR-TKI combined with angiogenesis inhibitors in NEJ 026 (9) and CTONG 1509 (8). Although patients were stratified by liver metastasis and EGFR mutation, there were still no data about efficacy of EGFR mutation with liver metastasis in immunotherapy combined with angiogenesis inhibitor and chemotherapy in IMpower150 (20). In general, liver metastasis is a negative predictor for EGFR-TKIs therapy in patients with EGFR-mutant NSCLC (21) without satisfactory combination therapies. In our study, the combination of EGFR-TKI and thymosin still showed no superiority. More samples were needed to confirm whether patients who received radiotherapy for CNS metastasis benefit from the combination therapy.

In the Iressa Pan-Asia Study (IPASS) (1), the common adverse events of gefitinib are rash or acne (66.2%), diarrhea (46.2%), dry skin (23.9%), and anorexia (21.9%). Erlotinib was reported to be the most prone to skin diseases in the first-generation of EGFR-TKIs (22). And the adverse events of icotinib were observed: rash (40%), diarrhea (19%), and hepatotoxicity (8%) (23). In our study, there was no statistical comparison of safety data in EGFR-TKI plus thymosin group and EGFR-TKI group. The most common adverse events were rash (40.0% in the EGFR-TKI plus thymosin group and 38.5% in the EGFR-TKI group), diarrhea (23.1% vs 25.4%), dry skin (20.0% vs 20.8%) and anorexia (9.2% vs 10.0%). It suggested that combination of EGFR-TKI and thymosin may be a safe option.

The inhibition of peripheral blood T cell subsets by EGFR-TKI therapy may be a potential reason why combined with thymosin can prolong PFS and OS. In this study, the number of CD3^+^T cells decreased significantly after treatment including both the CD3^+^CD4^+^T cells and CD3^+^CD8^+^T subsets decreased in the EGFR-TKI group. However, peripheral blood T cell subsets in the EGFR-TKI plus thymosin group did not change before and after treatment. It has been reported that erlotinib has the effect of immunosuppression while anti-tumor. Erlotinib could damage T-cell-mediated immune response through inhibiting T cell proliferation and activation, and inhibiting the secretion of IL-2 and IFN-γ by activated T lymphocyte cells (13). Treat with gefitinib for 4 weeks can result in a decreased percent of CD4^+^ T cells (24).

Thymosin, as a non-specific immunomodulator, had been widely used in cancer. It can increase the release of cytokine IL-2 and the expression of IL-2 receptor (25, 26). Thymosin was reported could not only activate T cells, inducing their maturation and differentiation (27), but also enhance the activity of natural killer and dendritic cells (28, 29). In addition, thymosin could enhance the expression of major histocompatibility complex class-I molecule and tumor associated antigens in multiple tumor cells (25, 30), thus tumor cells can be recognized by T lymphocytes easier. Several studies reported that both thymosin α1 and thymopentin could inhibit the growth of tumor cells by decreasing reactive oxygen species levels in tumor cells (31, 32). Together, thymosin plays important roles in both immunomodulatory and anti-tumor. Thus, the combination of EGFR-TKI with thymosin has synergistic effects in NSCLC.

In conclusion, our study revealed that combination of EGFR-TKI and thymosin can prolong the PFS and OS compared with EGFR-TKI monotherapy in NSCLC patients harboring active EGFR mutations without the increasing of adverse events. The combination therapy offers a new strategy for the treatment of NSCLC with active EGFR mutations.

## Data Availability Statement

The original contributions presented in the study are included in the **Supplementary Material**. Further inquiries can be directed to the corresponding authors.

## Ethics Statement

The studies involving human participants were reviewed and approved by Ethics Committee, Xinqiao Hospital, Army Medical University. The patients/participants provided their written informed consent to participate in this study. Written informed consent was obtained from the individual(s) for the publication of any potentially identifiable images or data included in this article.

## Author Contributions

YF and GZ: Conceptualization, Methodology, Validation, Formal analysis, Investigation, Data curation, Writing - original draft, Visualization. SL, PH, GL, FC, WZ, YD, and AZ: Data curation, Methodology, Validation. ZC: Conceptualization, Resources, Data curation, Writing - review and editing, Supervision, Project administration. JS: Conceptualization, Resources, Data curation, Writing - review and editing, Supervision, Project administration, Funding acquisition. All authors contributed to the article and approved the submitted version.

## Funding

This study was supported by the National Natural Science Foundation of China [No. 81672841, 81773245 and 81972858], the talent Foundation of Chongqing [cstccxljrc201910], the Cultivation Program for Clinical Research Talents of Army Medical University in 2018 [2018XLC1010]. 

## Conflict of Interest

The authors declare that the research was conducted in the absence of any commercial or financial relationships that could be construed as a potential conflict of interest.

## Abbreviations

AEs, adverse effects; AJCC; American Journal of Critical Care; CI, confidence interval; DCR, disease control rate; ECOG, Eastern Cooperative Oncology Group; EGFR, epidermal growth factor receptor; TKI, tyrosine kinase inhibitor; HR, hazard ratio; NSCLC, non-small cell lung cancer; ORR, objective response rate; OS, overall survival; PD, progressive disease; PFS, progression-free survival; PS, performance status; RECIST, Response Evaluation Criteria In Solid Tumors.
