# Supplementary figures and images for: The Efficacy and Safety of Epidermal Growth Factor Receptor Tyrosine Kinase Inhibitor Combined With Thymosin in Advanced Non-Small Cell Lung Cancer Patients Harboring Active Epidermal Growth Factor Receptor Mutations
Source: Front Oncol. 2021 May 28;11:659065. doi: 10.3389/fonc.2021.659065 (PMC8195272; doi:10.3389/fonc.2021.659065)

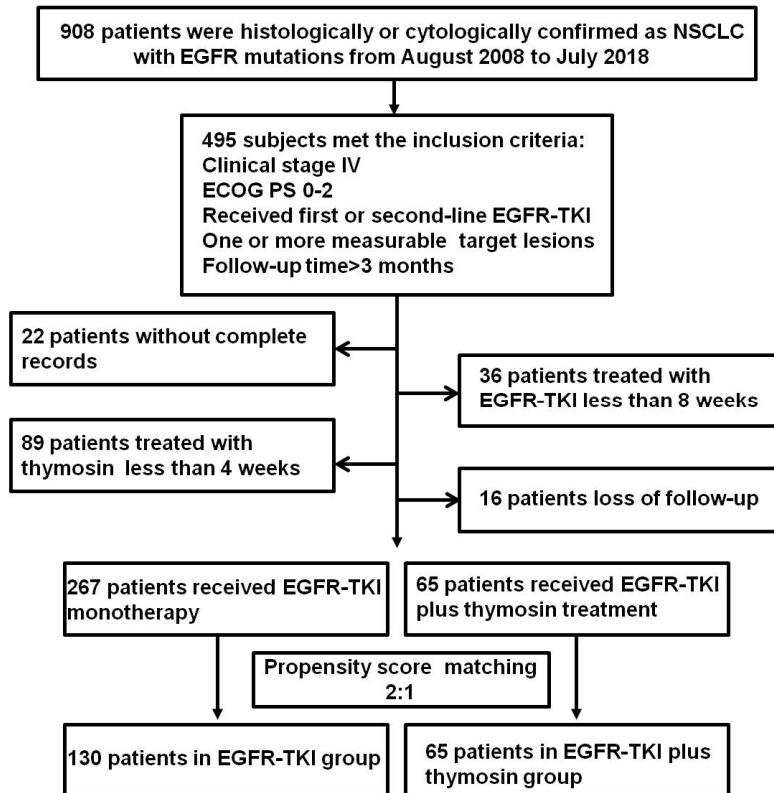

**Supplementary Figure 1. Study flowchart.**

Supplement: Supplementary file 1 [file DataSheet_1.pdf]
