# Supplementary material for: The Efficacy and Safety of Epidermal Growth Factor Receptor Tyrosine Kinase Inhibitor Combined With Thymosin in Advanced Non-Small Cell Lung Cancer Patients Harboring Active Epidermal Growth Factor Receptor Mutations
Source: Front Oncol. 2021 May 28;11:659065. doi: 10.3389/fonc.2021.659065 (PMC8195272; doi:10.3389/fonc.2021.659065)
Supplement: Supplementary file 2 [file DataSheet_2.pdf]

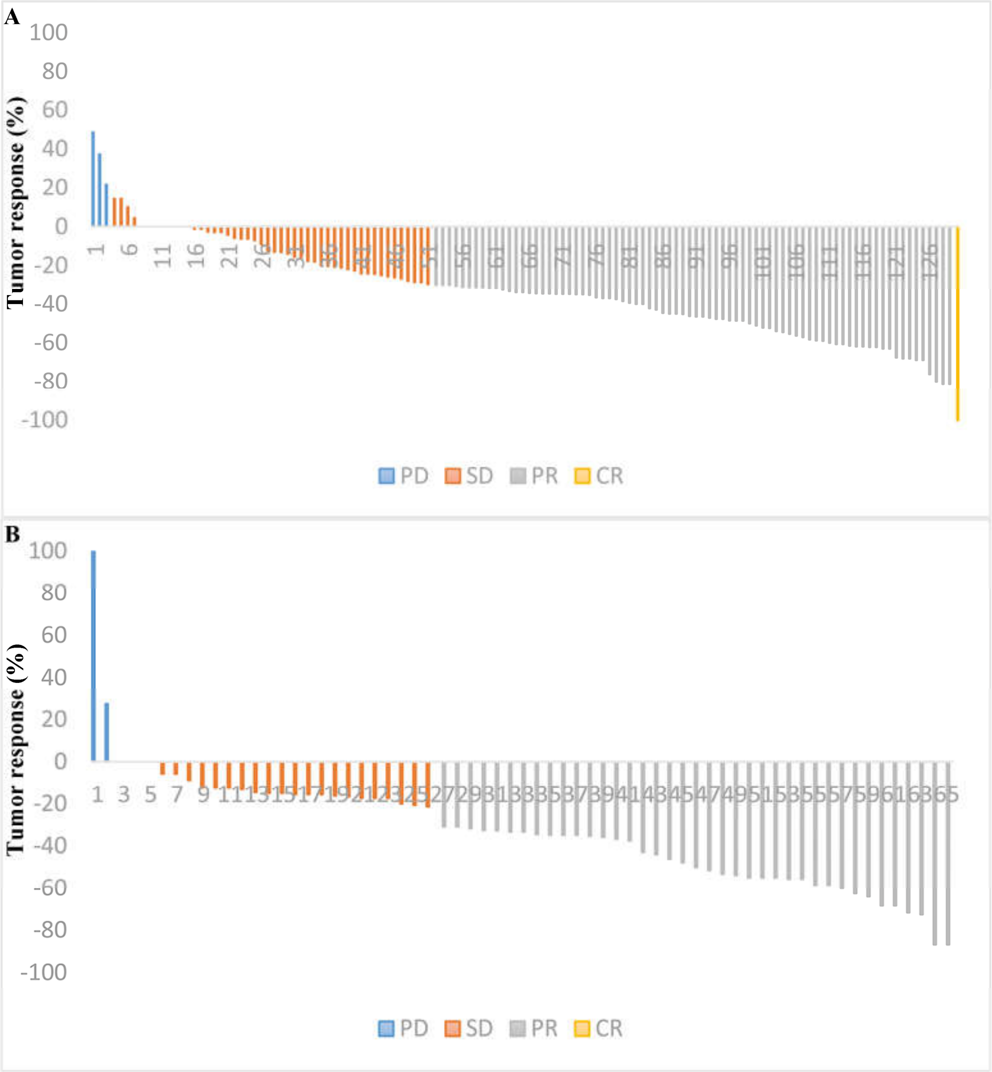

Supplementary Figure 2. Tumor response. Tumor response in EGFR-TKI group (A) and EGFR-TKI plus thymosin group (B). Abbreviations: PD, progressive disease; SD, stable disease; PR, partial response; CR, complete response.
